# Medical students’ perception towards mental health recovery: a descriptive qualitative study

**DOI:** 10.1080/10872981.2022.2106610

**Published:** 2022-07-27

**Authors:** Jonathan Han Loong Kuek, Ghee Kian Koh, Jenna Qing Yun OWYONG, Cyrus Su Hui Ho, Yong Shian Goh

**Affiliations:** a Susan Wakil School of Nursing and Midwifery, Sydney School of Nursing, Faculty of Medicine and Health, The University of Sydney, Australia; b Alice Lee Centre for Nursing Studies, Yong Loo Lin School of Medicine, National University of Singapore, Singapore; c Department of Psychological Medicine, Yong Loo Lin School of Medicine, National University of Singapore, Singapore

**Keywords:** Medical students, perception, mental health recovery, descriptive qualitative study, curriculum

## Abstract

The conceptualisation of recovery in mental healthcare, for which two definitions (‘clinical’ and ‘personal’) prevail, remains inconclusive. In most curricula of medical education, undergraduates are taught straightforward concepts of clinical recovery, which result in their perfunctory and rudimentary understanding. A qualitative descriptive approach was adopted to explore medical undergraduates’ perceptions of recovery for people with mental health conditions. Participants were recruited from a Singapore-based university through convenience sampling; the required sample size was determined by data saturation. Individual face-to-face interviews were conducted through Zoom, an online conferencing platform using semi-structured questions from March to July 2021. Recordings of the interviews were transcribed verbatim and thematically analysed. The Consolidated Criteria for Reporting Qualitative Research (COREQ) checklist guided the reporting of this study. Seventeen medical students with the majority (fifteen) of them in their fourth year of medical undergraduate education participated in this study. Four themes were identified: the relationship between mental illnesses and well-being; opinions on mental well-being; understanding of mental illnesses; and perceptions of recovery from mental illnesses. The medical undergraduates in this study predominantly conceptualised recovery based on functions, although many also agreed on respecting patients’ perspectives in defining it. This aligns with contemporary approaches that emphasise more shared decision-making opportunities and empowering people with mental health conditions. Accordingly, our findings highlighted the need for foundational medical education to incorporate these constructs in their curricula and strategise to provide more meaningful discussions about them.

## Introduction

Recovery in mental healthcare is often viewed through the lenses of the biomedical model where the emphasis remains on clinical recovery and symptom remission. Unfortunately, the complete remission of symptoms is often unattainable, in which case it may lead to a sense of failure and disappointment [1]. In the 1970s, there were efforts to expand this original biomedical model, resulting in the biopsychosocial model of care which emphasized the need for greater attention to be placed on psychological and social factors regarding mental health conditions [2]. Coupled with more patient and person-centered models of health and mental health care [3,4] that highlighted the need of a more humanistic perspective of care and support, with collaboration rather than direction being the core of services being provided. While these were positive steps forward and important milestones in medical education, clinicians were still in the driving seat and did not fully consider the lived experiences of the people they were supporting.

Thus, there has been a shift towards embracing the notion of recovery as a personal experience in mental healthcare [5]. Although the contemporary conceptualisation of personal recovery can be traced back to the late 1980s [6,7], its consumer-led movement has only gained momentum over the past decade, especially in Asia [8]. While the operationalisation of the term ‘personal recovery’ remains varied, one of the most commonly-cited definitions refers to it as ‘a way of living a satisfying, hopeful, and contributing life even with limitations caused by illness.’[6]

Recovery has been characterised as a complex and non-linear process [7] with numerous domains including hope, respect, empowerment, individuality, engagement in meaningful daily activities, and consumer involvement [9,10]. It is thus crucial to recognise that people experiencing mental health conditions (PMHCs) express a wide variety of recovery experiences. Such experiences are captured by the well-established CHIME framework through its five key processes: connectedness (good relationships with others); hope (optimism about the future); identity (positive sense of self); meaning (sense of meaning in life); and empowerment (sense of autonomy and control) [11].

Evidence from the literature over the past decade has supported the role of the recovery-oriented practices in optimising client outcomes, such as improved basic functioning and goal-planning, reduced legal involvement, and decreased rates and lengths of hospitalisation [12–14]. Hence, mental healthcare services are increasingly driven to incorporate recovery-oriented frameworks into their interventions [9]. Nonetheless, a drastic transition from the conventional biomedical model of care to a recovery-oriented model may be challenging for healthcare professionals. As a result, many tend to maintain a paternalistic control over care decisions [15]. Of note, this tendency is amplified in mental healthcare, since a diagnosis of mental illnesses may in extreme cases be sufficient grounds to act upon an individual without regard for their free will [16].

Furthermore, the stigma attached to PMHCs prevalent among the general public [17], healthcare professionals [18], and even the PMHC themselves [19] further hinders the successful implementation of the recovery-oriented model. Against this background, it is thus crucial for medical students to be sufficiently acquainted with the recovery-oriented modality, given their prospective roles as healthcare providers who may in future drive a transformation of mental healthcare [20]. Unlike countries within European and North American systems where more holistic perspectives of mental health care are considered the norm, in Singapore and most parts of Asia, there is still relatively less emphasis on mental health education [21,22] and based on what is known, there seems to be a greater focus on the biomedical aspects of mental health conditions, and coverage of biopsychosocial models of care and person-centred ones, in so far as to establish the complexity of mental healthcare and potential factors that influence the outcome.

While the incorporation of consumer involvement in the nursing curriculum has been highlighted in the literature [23], investigations into medical students’ knowledge of and attitudes towards personal recovery have been scarce, especially in an Asian country. Hence, this study aimed to explore medical undergraduates’ perceptions of recovery for PMHCs through the following research questions: (1) What are the medical undergraduates’ views on mental illnesses and mental well-being? (2) How do they perceive the concept of recovery in mental health? And (3) How do they derive their conceptualisations of recovery in mental health? The findings were envisioned to provide universities with insights into the undergraduates’ conceptualisations, attitudes, and understanding of recovery in the context of mental health and to identify and address knowledge gaps in the medical education system.

## Method

A qualitative descriptive approach [24] was used to explore the medical undergraduates’ perceptions of recovery among PMHCs. Additionally, the Consolidated Criteria for Reporting Qualitative Research (COREQ) checklist [25] guided the reporting in this study.

### Participants and setting

Participants were recruited from a Singapore-based university, which offers undergraduate and post-graduate medical degree programs. Each academic year, the faculty of medicine admits 300 medical students into their undergraduate medicine program. The participants for this study was recruited through purposive sampling. They were allowed to participate if they (1) were at least 18 years of age; (2) were existing medical students; (3) had not been formally diagnosed or self-diagnosed with psychiatric illness(es); (4) had not worked or was currently not working in a mental health facility (not including clinical placements); and (5) had no direct family members with mental health conditions. Additionally, the eventual sample size of this study was decided by data saturation [26] when interviewing more participants will not result in the identification of additional themes, ideas, opinions, or patterns during data analysis [27–29].

### Recruitment

Different platforms were used for recruitment: (1) announcements by the primary researcher after the undergraduates’ lectures, and (2) advertisements on the Department of Psychological Medicine’s Telegram messaging platform. Additionally, the research team’s contact details were provided on the recruitment emails and announcements for prospective participants.

### Data collection

Following our review of published works, four open-ended questions were formulated and sent to three experienced faculty members within the medical school to determine their clarity and relevance. Before the interviews, the participants were asked to complete a socio-demographic questionnaire consisting of eight multiple-choice questions. The interviews were then conducted in English over Zoom, an online video-conferencing platform. The primary researcher used open-ended questions such as ‘Tell me more about your views on what is recovery for people with mental health conditions’ to elicit the participants’ perceptions of recovery in the context of mental health. Field notes were also taken to aid the downstream transcription and data analysis of the key points elicited in the interviews [30]. Data collection lasted from March to July 2021, during which 17 interviews were conducted, each lasting up to 40 minutes.

### Ethical considerations

Ethical clearance was obtained from the Institutional Review Board of National University of Singapore (NUS IRB-2021-341) before the commencement of the study. All participants were briefed on the research aim, risks and benefits of participation, and their right to withdraw. No identifiers were collected during the study; therefore, anonymity was assured.

### Data analyses

The interviews were transcribed verbatim immediately after each session, thereby ensuring not only the veracity of transcription [31], but also a sense of awareness among the researcher team of their possible personal assumptions [32]. Two researchers independently used the six-step thematic analysis by Braun and Clarke [33] to analyse the data through systematic inductive coding and then finalised the themes. Subsequently, each theme was reviewed by the team such that the meanings derived from the data were accurately represented. The codes were rearranged when changes were made to them for their coherence with the respective themes. Finally, relevant verbatim quotes were extracted from the transcripts to support and better illustrate the themes [34].

### Rigour of findings

The methodological rigor of this study was ensured through the use of the criteria by Guba and Lincoln [35]. Firstly, member-checking was performed both by summarising the ideas expressed by the participants during the interviews and by returning the transcripts to them after the interviews. This step promoted better credibility of the transcription used in the data analysis [36]. Secondly, the contexts of the setting and participants in this study were outlined, as such details would enhance the transferability of our findings to the readers’ settings [26]. Lastly, our data collection and analysis were described in a transparent and accurate manner: this would enable not only future replication of this study, but also evaluation on the suitability of the decisions throughout the research [32].

## Results

### Participants’ characteristics

The participating medical undergraduates comprised 11 males and 6 females between 21 and 25 years of age (Table 1). The majority (15) of them were in their fourth year (medical undergraduate education in Singapore takes five years to complete). In terms of the participants’ clinical exposure, three of them had completed their psychiatric clinical rotation, four were in the midst of their rotation, another three were taking their psychiatric theoretical module, and the remaining seven had not had any formal psychiatric trainings or education exposures. Additionally, 12 of them had prior personal experiences with psychiatry beyond their formal education, through either their volunteer work or interactions with friends with psychiatric conditions.

**Table 1.** Demographics table.

Participant Code	Gender	Age	Year of Study	Have you taken any modules pertaining to mental health, psychiatry, or psychology within the school?	Have you completed the mental health clinical rotation?	Have you been exposed to mental health outside of school?
P1	Male	23	3	No	No	No
P2	Male	21	4	Yes; Psychiatry rotation	No	No
P3	Female	22	4	Yes; Psychiatry rotation	No	Yes
P4	Male	22	4	No	No	No
P5	Male	25	5	Yes; Psychiatry rotation	Yes	No
P6	Female	22	4	Yes; Psychiatry rotation	Yes	No
P7	Male	22	4	No	No	No
P8	Female	22	4	No	No	No
P9	Female	22	4	No	No	No
P10	Male	24	4	No	Yes	Yes
P11	Male	22	4	No	No	Yes
P12	Female	21	4	No	No	Yes
P13	Male	24	4	Yes; Psychiatry rotation	No	No
P14	Male	22	4	Yes; Psychiatry rotation	No	No
P15	Female	22	4	Yes; Psychiatry rotation	No	Yes
P16	Male	21	4	Yes; Psychiatry rotation	No	No
P17	Male	22	4	Yes; Psychiatry rotation	No	No

### Themes identified

Results from this study were categorised into four themes: the relationship between mental illnesses and well-being; the participants’ opinions on the construct of mental well-being; their understanding of mental illness; and their perceptions of recovery from mental illness. Each theme was further outlined as sub-themes and contributing factors.

### Relationship between mental illness and well-being

The first theme comprises two sub-themes on the multiplicity of views from the participants. Some of them have described mental illnesses and well-being as distinct concepts, while others have conceptualised them on the same spectrum.

### Mental illnesses and well-being as distinct concepts

Some participants shared their beliefs that mental illnesses and wellness were similar but separate concepts that could be present concurrently in a given individual. To illustrate this, one participant used the example of how symptoms of mood and anxiety disorders that did not fulfil clinical thresholds to constitute an illness could nonetheless compromise an individual’s well-being

… like, for example, if some guy has depression, so- this is how I try to think about it. So, let’s say he cannot find a job … . then now he is anxious to find a new job. I don’t think he has a relapse of his depression more of anxiousness due to stress which will affect his mental wellness, but it might not be a mental illness. – P17

However, others acknowledged that it would be challenging to define the impacts of mental illnesses. Whether mental wellness and illnesses could co-exist depended on the chronicity and fluidity of the mental health conditions. Despite their difference, the lines between wellness and illnesses were much less tangible and quantifiable.

But maybe in certain chronic mental conditions where it is well-controlled, one can still have the mental illness but has good mental well-being. If it is controlled. Ya. – P13

### Mental illnesses and well-being on a single spectrum

Most participants shared that mental illnesses and well-being existed on a single continuum, with illnesses being on the negative end and well-being on the positive one. This dichotomous view arose from the participants’ belief that the two could not co-exist, since the impact of the mental illnesses would preclude well-being.

… it’s quite hard, umm because once you have a mental illness likely your mental state is um, is affected, and you will not be in the right frame of mind; therefore, they cannot have like good mental well-being, that’s what I feel. – P7

### Opinions on mental well-being

The participants viewed the construct of mental well-being as a combination of two sub-themes: its holistic nature and its role as a preventive or protective mechanism against mental illness.

### More holistic in nature

The participants described well-being as being more all-encompassing than mental illness, associating it to multiple aspects of life rather than only experiences with a specific illness. For example, these could include daily routines and social connections.

Um, mental well-being, I would mean, to me, I feel like it would be more of like a holistic aspect of life so it’ll be like, your sleep, eating, daily living, feelings, and emotions. – P1

Additionally, well-being encompassed having greater insights over the general state of one’s mental health state, coping with challenges in life, and nurturing a positive outlook.

I’m not very sure also it’s just like my own personal idea like when we talk about mental well-being, I always think about like being positive and like uh, facing adversities and like using a positive mind set, yah. – P11

### A preventive or protective mechanism against mental illness

In addition to its holistic nature, mental well-being was viewed as a buffer against potential mental illnesses, even though the participants also acknowledged the caveat that it might not stop people from becoming mentally ill.

It seems to me mental well-being is more like preventive … it is something that one has to look out for and take care of like how we take care of our nutrition and exercise. – P13

One participant further shared how he believed that mental well-being could reduce the potential impacts of mental illnesses experienced by people.

… but I guess to me, yes to some degree in the sense that good mental well-being promote mental health and may reduce the occurrences of their mental conditions. – P16

### Understanding of mental illness

The participants’ understanding of mental illness, such as how they defined it and factors they perceived to cause these conditions, were grouped into three main sub-themes: the difficulty in defining mental illness; a biopsychosocial perspective; and functional impairment.

### Difficulty in defining mental illness

Quantifying and defining mental illnesses was a challenge for the participants, as most of them viewed them as complex and intangible. In particular, the lack of clear biological markers and casual factors were significant barriers for them to conceptualise mental illnesses.

So, for example like- from my understanding, like a brain tumour causing someone to act a certain way. It is something physical and quite easy to understand, I guess, causing some of the symptoms. Whereas in my mind, mental illnesses are abstract and more difficult to define. – P13

To find some semblance of concreteness amidst the ambiguity of what constituted a mental illness, some participants chose to rely on diagnostic criteria set by professional bodies, such as the Diagnostic and Statistical Manual of Mental Disorders (DSM) or International Classification of Diseases (ICD). This enabled them to have a framework with which to understand, diagnose, and make sense of mental illnesses.

Interviewer: I see, so in that sense right, how do you conceptualise a mental illness, is it just by the functioning or like, what makes a mental illness?

Participant: Mm, hard for me to say, because it’s like psychiatrists that define that, so my understanding is uh, uh based on those diagnostic criteria. – P5

### Biopsychosocial perspective

A consensus emerged from among the participants that mental illnesses were not merely uni-dimensional: they underlined the need to include biological, psychological, and social explanations to capture the complexity of mental illnesses adequately.

So like it can be affected by your biological processes, I think certain diseases will predispose you to mental illnesses. Whereas like it’s not just biological, like it can be also like social psychological factors that come into play, so ultimately I think it’s not like just one aspect that’s causing mental illness. – P11

Some of the participants viewed certain mental illnesses as biological, while others deemed them more psychosocial. Despite the differentiation, the participants still highlighted the importance of considering mental illnesses through a multi-faceted perspective, instead of regarding them as single-domain problems.

… some of them are more bio-based, like all the genetics and chemicals in the brains. Um, but I note that psychosocial plays a big part in some mental illnesses. For example, in mood disorders or eating disorders, um in schizophrenia, I think the psychosocial part of it is quite huge compared to let’s say autism where it is more genetic, yeah. – P3

### Functional impairment

Impairment in daily functions was one commonly raised indicator that enabled the participants to pinpoint the end of mental distress and the start of mental illnesses. With the onset of such impairment, they believed that the symptoms or challenges faced by the individual had sufficiently compromised his or her daily function for them to be considered as mental illnesses.

Participant: Mm, I think mental illness would be like severe psychological disorders that it impairs your daily functioning.

Interviewer: Mm, when you say functioning, what do you mean?

Participant: Mm, I think functioning can be considered like personal life, social life, um work life. – P8

### Perception of mental health recovery

The participants’ views on recovery in the context of mental health were categorised into three sub-themes: functionally defined, patient-defined, and symptom-defined.

### Functionally defined

A salient sign of recovery was the PMHCs restoring their daily functions and continuing with their life. Some participants opined that this clinically meant a return to the PMHCs’ pre-morbid level of functioning, where they could to an extent, ‘restart’ their lives after the onset of their condition.

So, recovery, we must first understand what the pre-morbid is. … . If this person is introverted initially and then diagnosed with anxiety or depression, you treat. You cannot expect the person to go back to become an extroverted person. I mean, they are introverted, to begin with. – P14

However, other participants held that recovery did not necessarily mean a restoration to the baseline level of functioning; instead, it could mean the attainment of a functional level deemed acceptable by the PMHCs.

Participant: Mmm, I think recovery would be like a return to, I won’t say return to normal baseline function, but to develop strategies, such that they can lead a functional kind of life.

Interviewer: Mm. What does functional life mean?

Participant: Functional, like getting out of bed and um brushing one’s teeth, bathing, that kind of thing or even going to work. – P9

### Patient-defined

In considering how recovery should be defined, the participants maintained that the patients’ view on recovery represented an important consideration that should be respected.

… but I guess ultimately it also dependent on the patient, like the patient wants to like uh be symptom-free and stuff, I guess we can tailor the treatment to towards that.” – P11

When prompted about potential disparities in ideologies of recovery between healthcare professionals and patients, the participants indicated that the corresponding strategy would depend on the magnitude of the differences. They expressed that they would attempt to understand and accept the patients’ personal preferences and also to reconcile such disparate perspectives.

Interviewer: What if your future patient says they disagree with what you think recovery is? How would you reconcile it?

Participant: I guess I would want to know how they view recovery? Yeah, then I can see how our perspectives are different and similar. – P13

However, the notable caveat was that such reconciliatory attempts appeared to be circumscribed by the patients’ expression of their intention to harm themselves or others.

I’ll consider seeing the patient as a whole and think if his views on treatment are okay. If it doesn’t, like put him or others in any harm, then I am alright to go ahead with the patient’s uh proposed treatments.”– P11

### Symptom-defined

Symptomatic improvement and fewer relapses were also deemed by the participants to be essential aspects of recovery. These were crucial in view of the trickle-down impacts arising from the symptoms of mental illnesses, such as increased medical costs and consumption of healthcare resources.

Um well, I guess like indicators that the person is doing better would be like improvement of symptoms and the reduction of relapses and readmissions. Like how much the family or the patient has to spend on medications or admission, yah. – P6

## Discussion

Our results have provided an insightful understanding of how medical undergraduates conceptualised mental illnesses, mental well-being, and the notion of recovery in mental health. From their perspective, mental well-being represented an all-encompassing construct that was separate from and closely related to mental illnesses. Mental well-being was considered to holistically include areas of psychological function unrelated to mental ill health. Furthermore, it was highlighted as a barrier against mental illnesses, playing a critical role in mitigating their adverse impacts. On the other hand, mental illnesses were more challenging to define, given their perceived intangible nature and their lack of quantifiable aspects. Accordingly, most of the participants defaulted to biopsychosocial explanations, established diagnostic criteria, or functional impairments as proxies to conceptualise mental illnesses [37]. These findings were independent of whether a student had completed their psychiatric rotation and had been exposed to people experiencing mental health conditions, indicating the need for a more precise delineation of these closely-related constructs, alongside a greater emphasis on them. These discussions on how mental illnesses and well-being can co-exist should be incorporated into undergraduate medical education, given their central importance to the effective treatment of PMHCs [38,39]. Additionally, such knowledge allows the medical undergraduates to be more well-rounded in their approach towards mental healthcare. For example, discussions could include earlier models of care such as biomedical, biopsychosocial, and person-centered ones and their limitations, before introducing how the personal recovery one fills in such gaps.

In terms of recovery, the participating medical undergraduates predominantly conceptualised it based on functions, although many of them also recognised the importance of the patients’ perspectives in defining it. Their responses evidently suggest a desire for a collaborative approach towards the care for PMHCs and management of their mental illnesses. This aligns with contemporary modalities that not only emphasise more frequent shared decision-making, but also focus more on empowering PMHCs [1,15] which seems to be taking it a step beyond earlier ideas regarding person-centered care. This is an encouraging finding, since it demonstrates a willingness among the undergraduates to move past traditional biomedical approaches and to embrace cooperation for a more meaningful recovery process for the PMHCs. In this context, evidence has suggested that the involvement of PMHCs in the pedagogical process can provide much-needed nuances in these discussions [23]. In addition, such an opportunity can provide preliminary experiences for the undergraduates to directly converse with PMHCs and understand their journey towards recovery, better aligning with how curriculums in countries where personal recovery is more emphasized are evolving.

Longitudinal studies on how the medical undergraduates’ attitudes change throughout their training will also provide critical insights into whether our findings will remain similar in the long term. Notable parallels can be drawn from a recent similar study on nursing students from the same medical school in Singapore [40]. In defining mental illnesses, the participants from both studies demonstrated a similar tendency to rely on biological symptoms and markers pertaining to the brain and established diagnostic criteria such as the DSM. However, in this study, the medical undergraduates included psychosocial factors in understanding the development of mental illnesses, which could be due to a strong focus on a biopsychosocial perspective that is currently being taught in their curriculum. In defining recovery in mental health, the participants from both studies commonly used the function-defined perspective, referring to the pre-morbid state as a yardstick for functionality. As with the medical undergraduates in this study, the nursing students expressed the sentiment that a return to the pre-morbid state would be unrealistic [40]. They also held that healthcare professionals should work with patients to determine recovery progress in aspects such as daily living while managing residual symptoms [40].

In conceptualising mental well-being, the participants from both studies commonly expressed the notion that mental illnesses and well-being existed on a continuum and that the two were mutually exclusive. Some nursing students also thought that mental well-being would aid recovery in mental health [40] though they made no mention on whether mental well-being could be used as preventive or protective buffer against mental illnesses [40], a role that was reported by the medical undergraduates in this study. As a whole, the similarities in findings between the two studies corroboratively underline areas in mental health education in Singapore’s medical schools that can be improved.

## Limitations and future directions

First, majority of our participants had not undergone their psychiatric rotations. Hence, their current views might have been limited by their lack of practical experiences in applying their beliefs and knowledge. Second, these views were solicited by interviewing them in the early stages of their careers, it is uncertain how their formal practice may evolve with increasing exposure to more experiences throughout their careers. Third, the study was conducted on a participants recruited from one Singapore-based university which may limit its transferability. However, the research team adhering closely to the criteria set by Guba and Lincoln ensured the transferability of the results. The comprehensive description of the research context and having the identified themes central to the research aims, allowed future scholars make independent informed judgment on the adequacy of the results in their respective setting. Finally, given the exploratory nature of our study, we did not focus on the potential influence of the school on participants’ perspectives on mental health recovery.

## Implications

Our findings have highlighted the need for a greater emphasis on the complexities and nuances of mental illnesses, especially regarding the conceptualisation of recovery and well-being. Therefore, foundational medical education should better incorporate these constructs within their curricula and strategise to promote meaningful discussions about them. Additionally, such discussions should consider the undergraduates’ existing beliefs, which may vary from person to person, as has been observed for the participants in this study. The participants in this study have had either academic or indirect personal exposure to mental health that allowed them to demonstrate some understanding of mental illnesses. Coupled with the expressed desire for communication and collaboration with PMHCs, this observation has revealed an absence of more mental health-related education and a growing interest in it. In this regard, it is important to note that existing clinical placements are predominantly psychiatric postings, with a primarily biological focus on treatment and recovery. Accordingly, varying the clinical placements to include institutions beyond the traditional hospital setting may provide the undergraduates with more holistic experiences with PMHCs [41].

## Conclusion

Against the background of evolving conceptualisation of mental illnesses, well-being, and recovery, undergraduate medical education continues to develop. Our findings evidently suggest that there are gaps in foundational medical education in relation to how knowledge of mental illnesses and well-being is imparted. Tertiary education and clinical placements represent crucial channels for medical undergraduates to obtain knowledge and experiences in mental healthcare. These channels therefore present an opportunity to further bolster their foundational knowledge beyond the traditional clinical or biological understanding of mental illnesses and, by extension, their perspectives on recovery. As health is not complete without mental well-being, medical professionals need to be familiar with such concepts to deliver more holistic mental healthcare services. Hence, the undergraduate education of prospective medical professionals should place a greater emphasis on these concepts.

## Data Availability

The datasets used and/or analysed during the current study are available from the corresponding author on reasonable request.
